# Prolonged progression‐free survival achieved by octreotide LAR plus transarterial embolization in low‐to‐intermediate grade neuroendocrine tumor liver metastases with high hepatic tumor burden

**DOI:** 10.1002/cam4.4628

**Published:** 2022-03-14

**Authors:** Yiming Liu, Haikuan Liu, Wenchuan Chen, Hang Yu, Wang Yao, Wenzhe Fan, Jiaping Li, Minhu Chen, Jie Chen, Yu Wang

**Affiliations:** ^1^ Department of Interventional Oncology, the First Affiliated Hospital Sun Yat‐sen University Guangzhou China; ^2^ Department of Gastroenterology, the First Affiliated Hospital Sun Yat‐sen University Guangzhou China

**Keywords:** embolization, therapeutic, liver, neoplasm metastasis, neuroendocrine tumors, octreotide LAR

## Abstract

**Objective:**

To evaluate the efficacy and outcome of transarterial embolization (TAE) plus octreotide long‐acting repeatable (LAR) on patients with low‐to‐intermediate neuroendocrine tumor liver metastases (NETLM).

**Methods:**

One hundred and sixteen patients with G1/G2 NETLM treated with TAE plus octreotide LAR at the First Affiliated Hospital, Sun Yat‐sen University between January 12, 2016 and September 24, 2020 were reviewed. Radiological response was evaluated according to response evaluation criterion in solid tumor version 1.1. Overall progression‐free survival (PFS) was assessed. Intrahepatic and extrahepatic PFS were evaluated in the whole cohort and in patients with the extrahepatic disease (EHD), respectively. Factors affecting treatment response and overall PFS were analyzed using the logistic regression model and Cox proportional hazard model. Adverse events were recorded and evaluated according to Common Terminology Criteria for Adverse Events 5.0.

**Results:**

The median overall PFS of the whole cohort was 13.6 months. For the patients with EHD, the median intrahepatic PFS and extrahepatic PFS were 13.6 and 26.1 months, respectively. The median overall PFS of patients with hepatic tumor burden (HTB) <10%, 10%–25%, 25%–50%, and >50% were 25.2, 13.6, 11.2, and 12.3 months, respectively. Ki67 >10%, HTB >50%, and bone metastasis were independently associated with overall PFS. The objective response rate was 78.4%. In patients with HTB 25%–50% and >50%, responders (complete response or partial response) had significant prolonged PFS compared with nonresponders (stable disease or progression disease). Ki67 >10%, bone metastasis, and clear tumor margin were independently associated with response to TAE. The most frequent adverse events that occurred after TAE were postembolization syndrome, and no treatment‐associated death occurred during the perioperative period.

**Conclusion:**

Transarterial embolization plus octreotide LAR can significantly prolong the PFS of neuroendocrine tumor liver metastases, especially with high HTB over 50%. Selected patients with HTB >25% (ki67 ≤10%, absence of bone metastasis, clear tumor margin) could derive prognostic advantage from the combined treatment.

## Graphical abstract

TAE plus octreotide LAR can prolong PFS for patients with NETLM. Patients with high hepatic tumor burden and without bone metastasis might have a better prognosis from combined treatment of TAE and octreotide LAR.

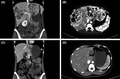

## 1 INTRODUCTION

Neuroendocrine neoplasms (NENs) are a group of heterogeneous tumors originating from neuroendocrine cells.^1^ Based on their proliferative ability, the World Health Organization (WHO) categorizes the well‐differentiated neuroendocrine tumor (NET) into three grades according to Ki‐67 percentage and mitotic index.^2^ Grade 1 (G1) and G2 NET, also known as low‐to‐intermediate grade NET, are those with a Ki‐67 ≤20% and mitotic index ≤20/high power field, normally presenting an indolent nature.^2^ However, distant metastases could be found in a majority of NET patients either at diagnosis or during therapy, resulting in a relatively worst prognosis. Common metastatic sites for NETs include liver, lymph node, peritoneum, bone, etc., and the liver is the most frequent site.^3^, ^4^ The presence of liver metastases was linked to poor prognosis, and the survival outcome of patients with neuroendocrine tumor liver metastases (NETLM) could be compromised with increasing extent of hepatic tumor burden (HTB).^5^, ^6^, ^7^ NETLM were generally categorized into three types according to their distribution for the purpose of selecting candidates of surgical therapy, and a majority of patients with NETLM present with type III liver metastases, referring to as “disseminated spread, both liver lobes involved.”^8^

Somatostatin analogs (SSAs) are a well‐established therapy in metastatic NETs, based on their ability in both symptom control and antiproliferation. The PROMID study demonstrated that octreotide long‐acting repeatable (LAR) prolonged time to tumor progression (TTP) and found a tendency that patients with low HTB at initial treatment had a survival advantage when treated with octreotide LAR.^7^, ^9^ The CLARINET study found that prolongation of progression‐free survival (PFS) could be achieved in advanced NET patients with prior stable disease (SD) being treated with lanreotide with median PFS of 32.8 months versus placebo PFS 14.0 months.^10^, ^11^ The ESMO guideline has placed SSAs in the first‐line therapy for G1/G2 metastatic NETs with positive somatostatin receptor (SSTR) expression.^12^ However, in the PROMID study, the median TTP in the octreotide LAR group for those with HTB more than 50% was only 4.6 months, suggesting that the effect of SSA was poor in this type of patient. According to the result of PROMID study, overall survival in patients with HTB more than 10% was significantly shorter than those patients with HTB ≤10% (106 months vs. 58 months). Therefore, the influence of HTB on PFS and overall survival of PROMID study speculated that if additional therapeutic methods such as TAE could reduce the HTB rapidly and effectively, prolonged PFS might be achieved by SSA.

Given the large proportion of patients with bilobar involvement, surgical resection and ablation could only be performed on a minority of highly selective NETLM patients.^8^ Liver‐directed embolotherapies including transarterial embolization (TAE), chemoembolization (TACE), and radioembolization (TARE) are considered effective alternatives to surgical approach, with reported objective response rates (ORR) up to 72%.^13^, ^14^, ^15^, ^16^, ^17^, ^18^ Despite the lack of relevant controlled study, embolotherapies have shown promising long‐term outcomes in NETLM patients and are widely regarded as beneficial to a large group of patients' prognoses.^17^, ^19^ However, the regimens of systemic therapy, which were the basic treatments for metastatic NETs, were not uniform in the study cohorts in most previous studies concerning embolotherapy, introducing potential confounding factors into the studies. We previously studied the safety and treatment response of the combined treatment with TAE plus SSA on NETLM and found that the combined regimen can safely and effectively reduce HTB.^20^ Yet, the previous study only focused on the evaluation of ORR and did not assess the prognostic outcome such as PFS due to the small sample size and limited follow‐up time. The long‐term outcome of TAE plus SSA in managing NETLM, especially those with a high tumor burden, has rarely been reported. In this context, we conducted the present retrospective study, reviewing low‐to‐intermediate grade NETLM cases treated with TAE plus octreotide LAR in our center. We aimed to (1) assess the efficacy and long‐term outcome of TAE plus octreotide LAR in managing NETLM, especially with high HTB, and (2) distinguish which group of patients could obtain benefit from the treatment with TAE plus octreotide LAR.

## 2 PATIENTS AND METHODS

### 2.1 Study design and case enrollment

**FIGURE 1:**
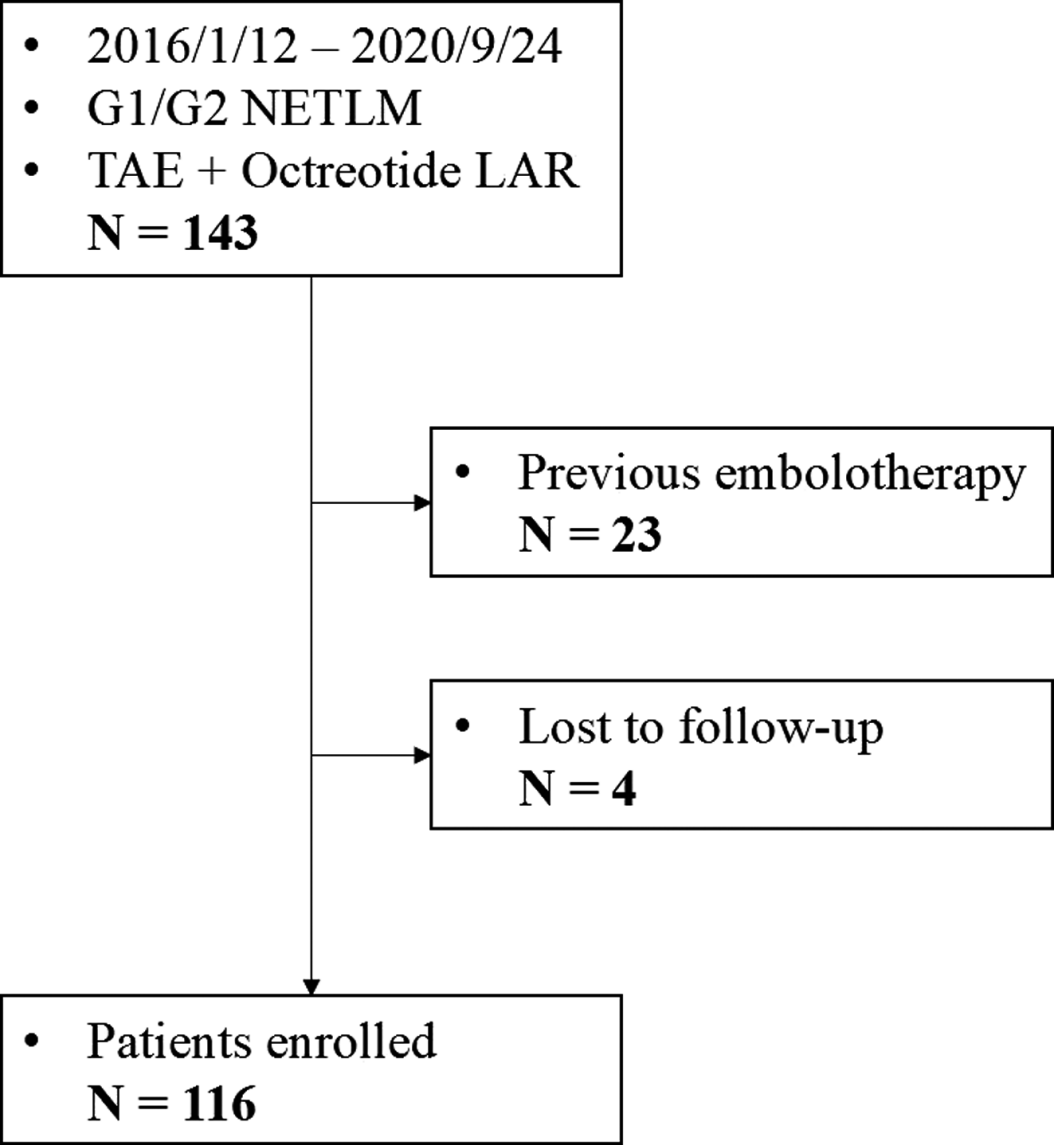
Flow chart of cohort enrollment

This was a single‐arm, retrospective cohort study performed at the First Affiliated Hospital of Sun Yat‐sen Hospital. The study was approved by the Ethics Committee of the First Affiliated Hospital of Sun Yat‐sen Hospital (Ethical number: [2021]823). Consecutive cases between January 12, 2016 and September 24, 2020 were enrolled according to the following criteria: (1) histologically proven G1/G2 NET with metastatic liver disease, (2) having undergone complete TAE sessions, and (3) being treated with octreotide LAR (Sandostatin‐LAR; Novartis) as combined systemic therapy. Cases were excluded if they (1) previously underwent embolotherapy in other hospitals, (2) were lost to follow‐up after TAE treatment, (3) could not tolerate the operation due to poor cardiopulmonary, liver, and kidney function, and (4) combined application of other antitumoral medication. Figure 1 shows the flow chart of patient enrollment.

### 2.2 Treatment

The therapeutic regimen for all NET patients in our center was made based on the opinion of a multidisciplinary team (MDT). Before using octreotide LAR, ^68^Ga‐DOTATATE‐PET/CT and immunohistochemical tests were performed to confirm positive SSTR expression on the tumors. Octreotide LAR was intramuscularly injected with an initial dosage of 30 mg and a frequency of 28 days.^9^ The decision of adjusting dosage was made by the MDT taking into consideration of the patients' hormone‐related symptoms, tumor classification, and treatment response. TAE procedures were simultaneously performed at the beginning of octreotide LAR administration if patients met the following indication: (1) predominant liver disease not eligible for resection or ablation, (2) ECOG score ≤ 2, (3) Child‐Pugh score A or B, and (4) absence of biliary duct dilation or cholangitis.

All TAE procedures were performed in the context of obtaining informed consent from the patients and their families. To prevent the potential threat of carcinoid crisis during TAE, the patients were started on continuous intravenous infusion of octreotide before the procedure. Patients were under local anesthesia. Access to the femoral artery was obtained by Seldinger's technique, followed by guiding a 5F catheter to celiac trunk and superior mesenteric artery, respectively, in which angiographies were carried out to visualize hepatic arterial anatomy, tumor blood supply, and portal vein patency. Further catheterization was performed to reach tumor‐involved lobar or segmental arterial branches, and 40–120 μm Embosphere (Merit Medical) and 100 μm polyvinyl alcohol (Cook) were injected into the microcatheter in sequence under fluoroscopy. Embolization ceased when the intra‐arterial contrast agent column was visible at the tip of the microcatheter within two to five heartbeats. All procedures are the same as in our previous report.^21^ Three sessions with an interval of 4–6 weeks were performed unless enhanced area in lesions were totally disappeared or there was any progressive disease (PD) found in preprocedural imaging scans.^21^

### 2.3 Data collection

**FIGURE 2:**
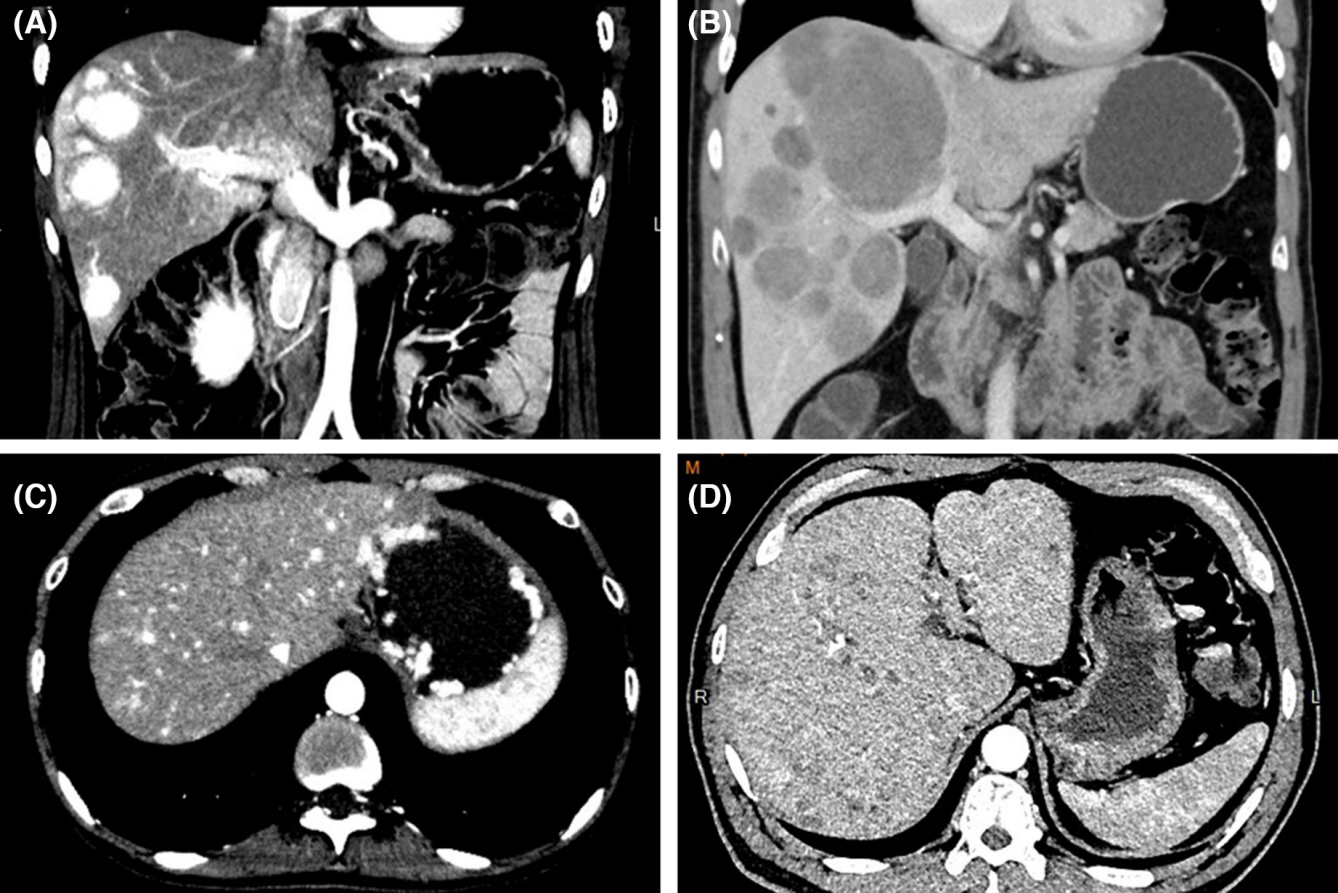
Examples of various characteristics of neuroendocrine tumor liver metastases shown in contrast CT image, including hypervascular (A, C), hypovascular (B, D), with clear tumor border (A, B) and without clear border (C, D)

Multiphasic contrast cross‐sectional imaging, ^18^F‐FDG, and ^68^Ga‐DOTATATE PET/CT of the whole body were run at baseline. Contrast CT/MR of the upper abdomen was repeated 1 month after each TAE session. After the completion of TAE treatment, cross‐sectional imaging was carried out with an interval of 3–6 months to monitor the disease status. The radiological response was judged independently by a third‐party evaluation group according to response evaluation criteria in solid tumor version 1.1 (RECIST 1.1).^22^ In the case that not all hepatic lesions were treated in 1 TAE session, only the treated lesions were assessed by RECIST 1.1. Radiological features of hepatic lesions were evaluated on the baseline image by 2 experienced radiologists blind to the treatment outcome. Arterial phase enhancement was defined as a significant hyperattenuation of the lesion compared with the adjacent liver parenchyma by a visual assessment on the arterial phase.^23^ Clear margin was defined as a well‐defined border between the lesion and the liver parenchyma by the visual assessment on the arterial or portal phase. Figure 2 shows the example for various characteristics mentioned above. The HTB was assessed from 4 to 6 slices of a CT/MR scan by a semiquantitative 3‐dimensional approach and categorized as <10%, 10%–25%, 25%–50%, and >50%.^6^, ^9^, ^24^

Laboratory tests, including liver enzymes, serum albumin, total bilirubin, prothrombin, etc. were systematically obtained within 3 days before and after each TAE session and at each subsequent follow‐up. Adverse events were assessed and recorded according to Common Terminology Criteria for Adverse Events version5.0.

### 2.4 Outcomes

The primary endpoint of the present study was PFS, defined as the time period from initial TAE to the first presence of PD in any organ or all‐cause mortality. Intrahepatic PFS and extrahepatic PFS were assessed, respectively, according to the specific location of tumor progression. Intrahepatic PFS and extrahepatic PFS were additionally evaluated in the patients with the extrahepatic disease (EHD), which was defined as patients with extrahepatic metastases or/and unresected primary tumors. The secondary endpoint was ORR, defined as the proportion of patients achieving complete response (CR) or partial response (PR) as the best response to TAE according to RECIST 1.1.

### 2.5 Statistical analyses

Statistical analyses were performed using SPSS software (Version 23.0; IBM Corp.). Statistical figures were made using R software (Version 4.0.3). Continuous variables were described by median and range and compared using the Mann–Whitney *U* test. Categoric variables were described by frequency and percentage and compared using the chi‐square test. Kaplan–Meier method was employed to analyze survival data and the comparison was performed using the log‐rank test. Univariate and multivariate logistic regression models were used to analyze factors affecting treatment response. Univariate and multivariate proportional hazards Cox regression models were applied to analyze factors affecting overall PFS. Factors with a *p* value of <0.10 in univariate analysis were included in the multivariate model. Differences with *p* < 0.05 were considered statistically significant.

## 3 RESULTS

### 3.1 Patients

One hundred and forty‐three cases met the inclusion criteria and were preliminarily included. Twenty‐three were excluded for previous TAE/TACE treatment in other hospitals and four were excluded for loss to follow‐up after TAE treatment in our center. A total of 116 cases were finally enrolled in our study cohort (Figure 1). The total number of TAE procedures performed was 291, with an average number of it per patient of 2.5. The baseline characteristics were summarized in Table 1. Patients with G2 tumors accounted for 86.2% and most were with a ki67 index ranging from 3% to 10%. The pancreas (46.6%) was the most common primary site, followed by the rectum (31.0%). In six patients, the primary site was unknown. The primary tumor was resected in 45 patients (38.8%). Sixteen patients had a treatment history of ablation or surgery for liver metastasis. Approximately 60% of patients were found with extrahepatic metastasis. Fifty‐eight patients (50.0%) had lymph node metastasis and 30 (25.9%) had bone metastasis.

**TABLE 1:** Baseline characteristics of patients

	Mean (range)/*N* (%)
Age	52 (18–73)
Gender	
Male	54 (46.6)
Female	62 (53.4)
Primary site	
Pancreas	54 (46.6)
Rectum	36 (31.0)
Intestine	14 (12.1)
Stomach	5 (4.3)
Lung	1 (0.9)
Unkown	6 (5.2)
Ki67	8 (1–20)
<3%	16 (13.8)
3%–10%	82 (70.7)
>10%	18 (15.5)
Liver involvement	
<10%	31 (26.7)
10%–25%	26 (22.4)
25%–50%	24 (20.7)
>50%	35 (30.2)
Extrahepatic metastasis	74 (63.8)
Bone	30 (25.9)
lymph node	58 (50.0)
Lung	5 (4.3)
Others	14 (12.1)
Extrahepatic disease^a^	100 (86.2)
Previous treatment for Liver Metastases	
Ablation	6 (5.2)
Surgery	8 (6.9)
Surgery + ablation	2 (1.7)
Primary tumor resection	45 (38.8)

^a^ Extrahepatic disease includes extrahepatic metastases and unresected primary tumor.

### 3.2 Outcomes

**FIGURE 3:**
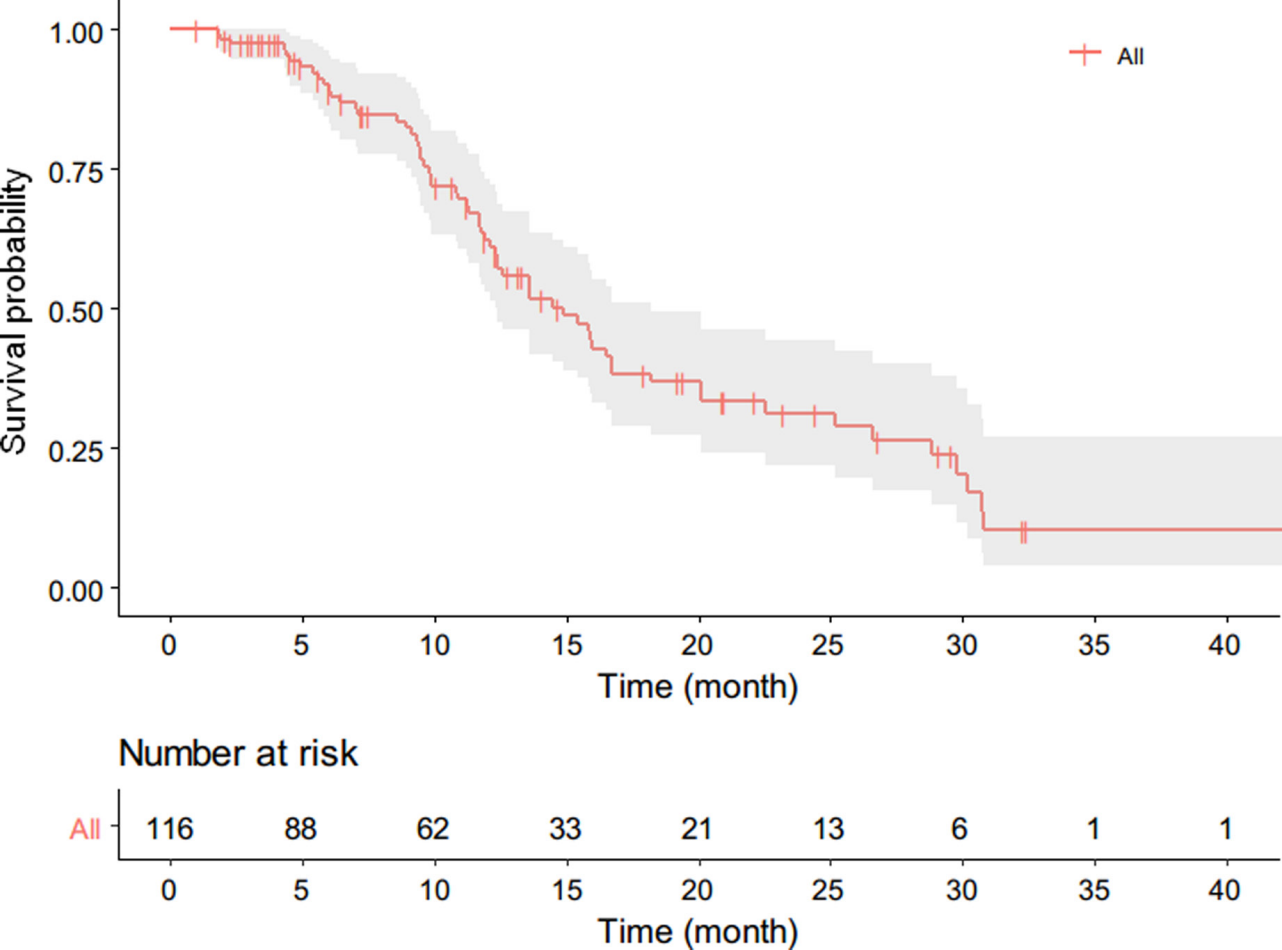
Kaplan–Meier curves of overall PFS for the whole cohort

**FIGURE 4:**
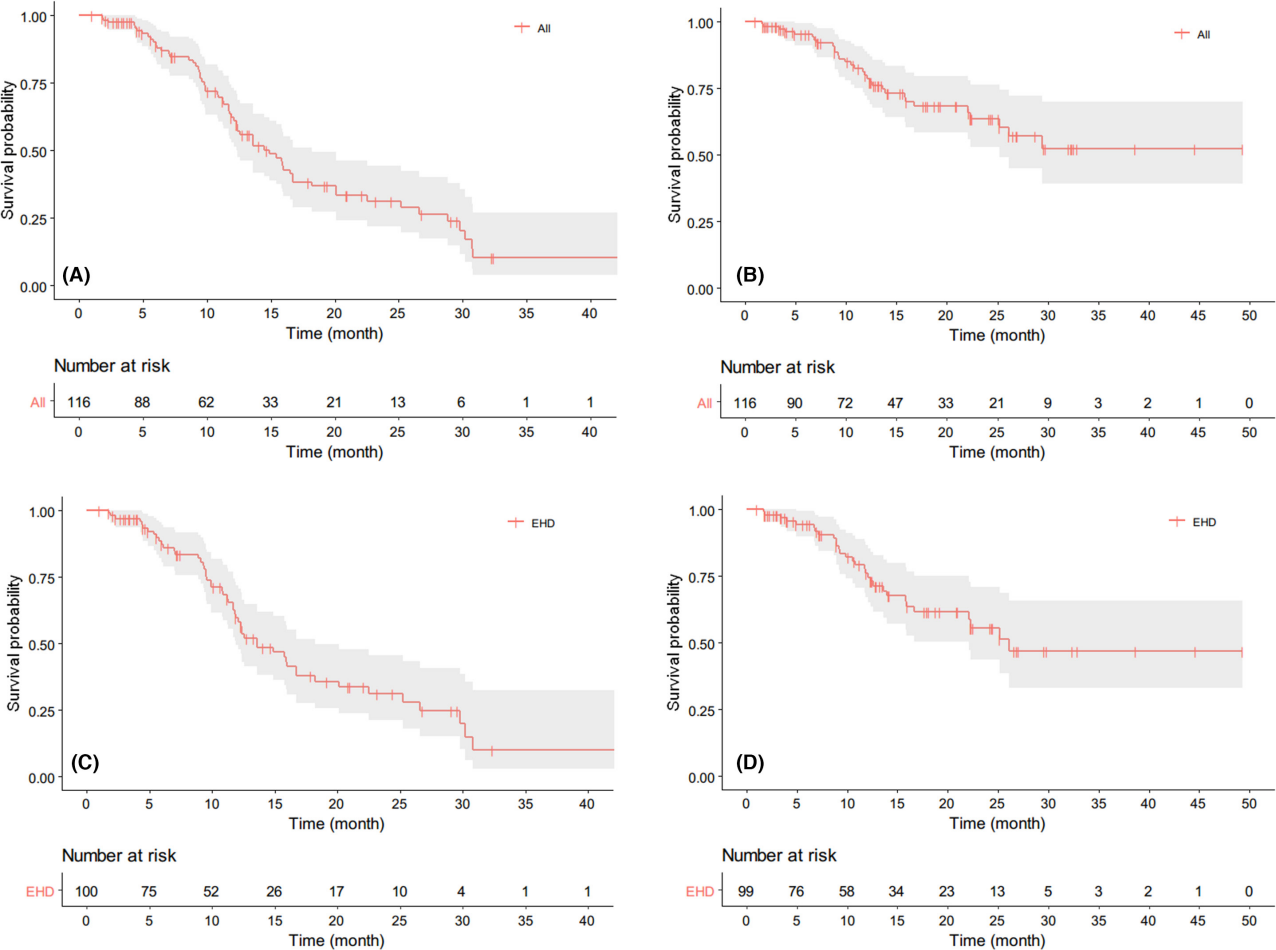
Kaplan–Meier curves of intrahepatic progression‐free survival (PFS) (A) and extrahepatic PFS (B) for the whole cohort; intrahepatic PFS (C) and extrahepatic PFS (D) for the patients with extrahepatic disease (EHD). The median PFS was 14.9 months (A), not reached (B), 13.6 months (C) and 26.1 months (D), respectively

**FIGURE 5:**
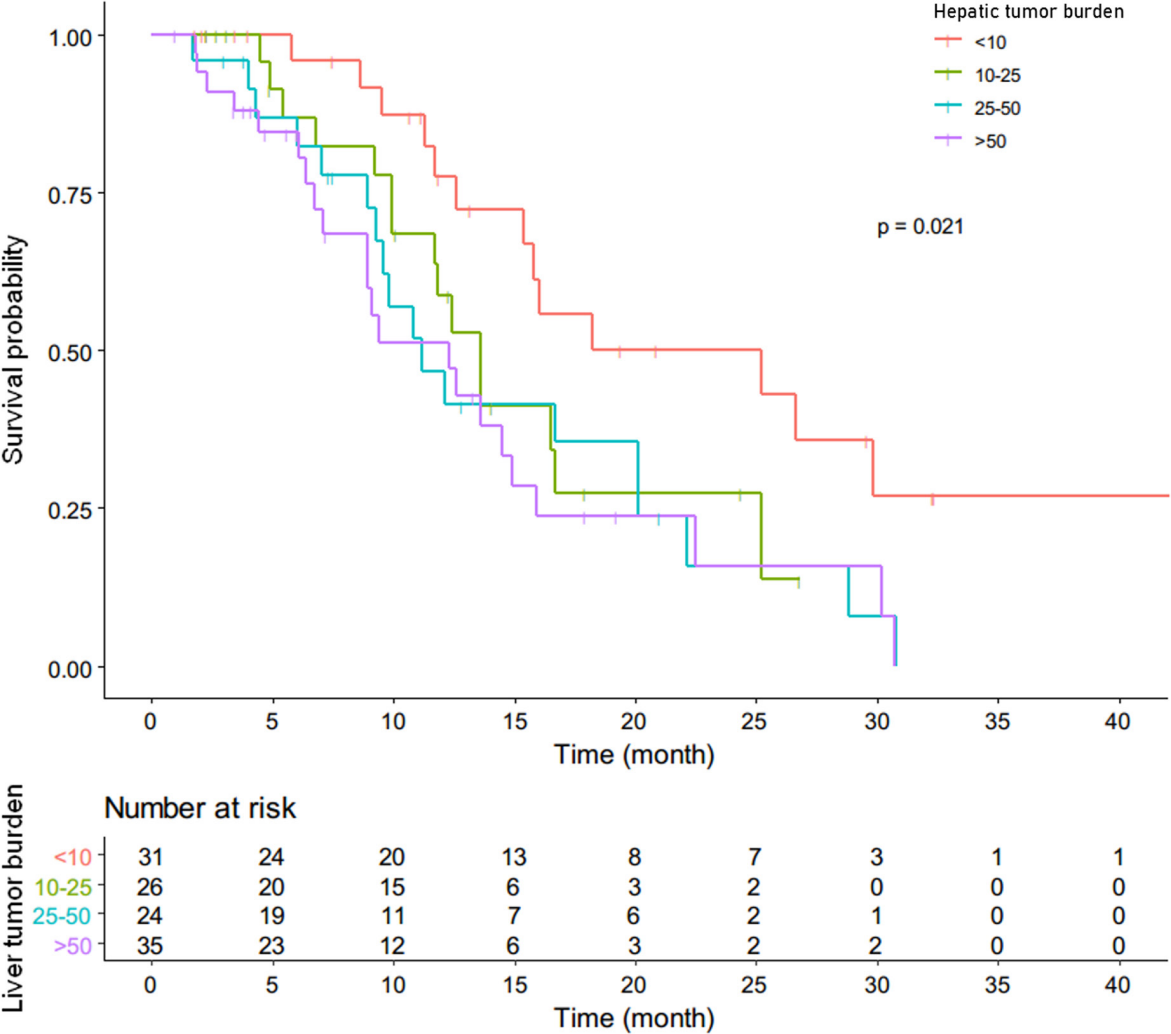
Kaplan–Meier curves of overall PFS in different groups according to hepatic tumor burden

**FIGURE 6:**
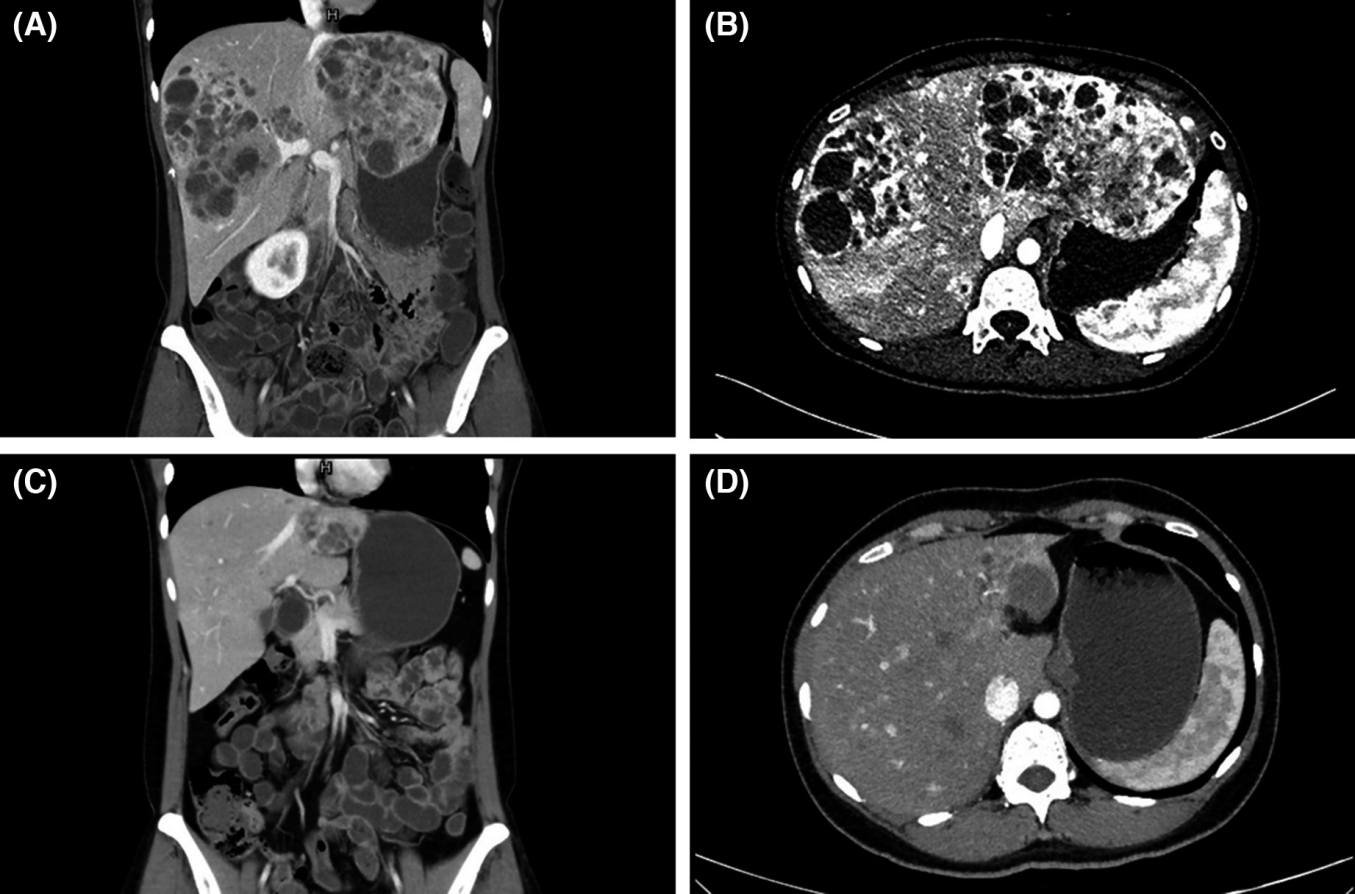
Multiple neuroendocrine tumor liver metastases with unknown primary site were found in a 37‐year‐old female patient (A, B). The ki‐67 was 10%. Three sessions of transarterial embolization (TAE) were performed. Administration of octreotide LAR with a dosage of 30 mg/4 weeks was started simultaneously. Reassessment was performed 3 months after TAE. Partial response by response evaluation criterion in solid tumor (RECIST) and complete response by modified RECIST were achieved (C, D). Progression disease has not been observed and the progression‐free survival of this patient was 26.8 months

The median follow‐up time of this study was 16 months with a range from 1 to 49 months. For the whole cohort, the median PFS was 13.6 months (Figure 3), the median intrahepatic PFS was 14.9 months, and extrahepatic PFS was not reached, respectively (Figure 4). For the patients with EHD, the median intrahepatic PFS and extrahepatic PFS were 13.6 and 26.1 months, respectively (Figure 4). The median PFS of patients with HTB <10%, 10%–25%, 25%–50%, and >50% were 25.2, 13.6, 11.2, and 12.3 months, respectively (Figure 5). According to RECIST 1.1, CR in 7 patients (6.0%), PR in 84 patients (72.4%), SD in 22 patients (19.0%), and PD in 3 patients (2.6%) were observed (Table 2). The ORR was 78.4%. An example of treatment efficacy was shown in Figure 6.

**TABLE 2:** Radiology response according to response evaluation criterion in solid tumor version 1.1

	*n* (%)
Complete response	7 (6.0)
Partial response	84 (72.4)
Stable disease	22 (19.0)
Progression disease	3 (2.6)

### 3.3 Factors affecting overall PFS

In univariate Cox regression, HTB and bone metastasis were significant factors. Ki67 was also brought into multivariate analysis because the *p* value was <0.1. In the multivariate Cox regression model, ki67 >10% (HR = 2.153, *p* = 0.048), HTB >50% (HR = 2.840, *p* = 0.003), and bone metastasis (HR = 2.911, *p* < 0.001) were independent risk factors affecting overall PFS (Table 3).

**TABLE 3:** Univariate and multivariate cox regression analyses for overall progression‐free survival

	Univariate			Multivariate		
	HR	95% CI	*p*	HR	95% CI	*p*
Age	1.009	0.987–1.032	0.420			
Gender						
Male	Ref					
Female	0.874	0.540–1.414	0.584			
Functionality						
No	Ref					
Yes	1.380	0.720–2.647	0.332			
Primary site						
Pancreas	Ref					
Nonpancreas	0.847	0.521–1.379	0.505			
Primary tumor resection						
No	Ref					
Yes	1.313	0.813–2.120	0.265			
Ki67						
≤10%	Ref			Ref		
>10%	1.981	0.966–4.063	0.062	2.153	1.006–4.607	0.048*
Hepatic tumor burden						
<10%	Ref			Ref		
10%–25%	2.022	0.945–4.322	0.069	2.087	0.956–4.554	0.065
25%–50%	2.425	1.182–4.974	0.016*	1.864	0.888–3.912	0.100
>50%	2.781	1.386–5.580	0.004*	2.840	1.410–5.720	0.003*
Extrahepatic metastasis						
Bone	3.539	1.978–6.333	<0.001*	2.911	1.526–5.554	0.001*
Lymph node	1.281	0.790–2.078	0.315			
Lung	0.777	0.279–2.161	0.629			
Others	1.067	0.458–2.482	0.881			
Hepatic tumor margin						
Unclear	Ref					
Clear	0.665	0.380–1.131	0.129			
Arterial phase enhancement						
Without	Ref					
With	0.712	0.435–1.166	0.177			

* Indicates the significant differences of the results in Univariate and multivariate proportional hazards Cox regression models which were applied to analyze factors affecting overall PFS. Bone metastasis and hepatic tumor burden 25–50%, >50% were factors with *p* < 0.10 in univariate analysis which included in the multivariate model. Ki‐67 >10%, Bone metastasis and hepatic tumor burden >50% were founded differences with *p* < 0.05 were considered statistically significant.

**FIGURE 7:**
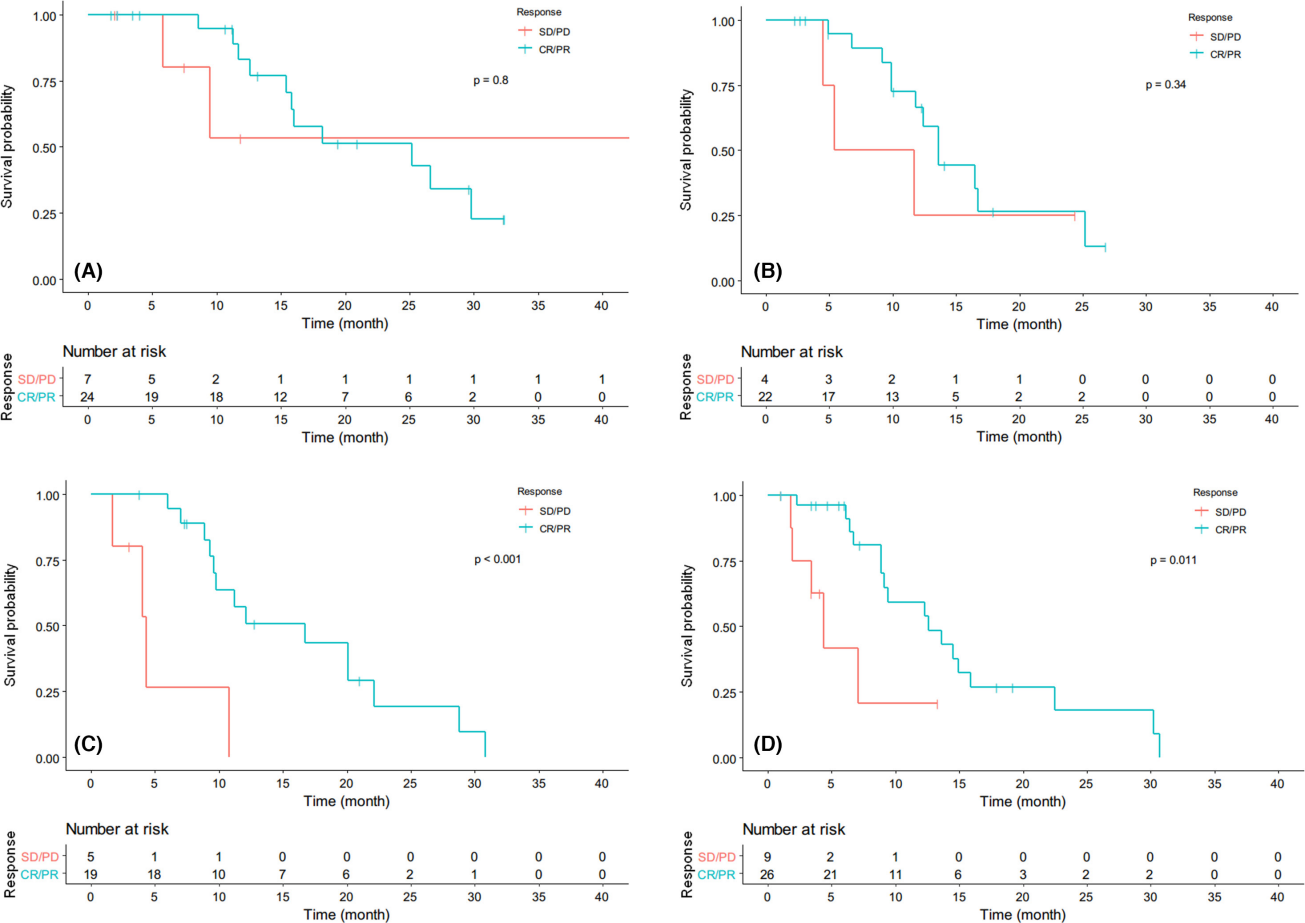
Comparison of overall progression‐free survival between different responses to transarterial embolization in patients with hepatic tumor burden <10% (A), 10%–25% (B), 25%–50% (C), and >50% (D), respectively. CR, complete response; PD, progressive disease; PR, partial response; SD, stable disease

The effect of treatment response on PFS was analyzed independently in the four subgroups according to HTB. In patients with HTB <10% and 10%–25%, there were no significant differences observed between responders (CR/PR) and nonresponders (SD/PD) (HTB <10%: *p* = 0.80; HTB 10–25%: *p* = 0.34), whereas in patients with HTB 25%–50% and >50%, responders had significant prolonged PFS compared with nonresponders (HTB 25%–50%: 16.7 months vs. 4.3 months, *p* <0.001; HTB >50%: 12.6 months vs. 4.4 months, *p* = 0.011) (Figure 7).

### 3.4 Factors affecting treatment response

In univariate logistic regression, ki67 >10%, bone metastasis, and clear tumor margin were significant factors. Primary site was also brought into multivariate analysis because the *p* value was <0.1. In the multivariate logistic regression model, ki67 (OR = 0.181, *p* = 0.01) and bone metastasis (OR = 0.149, *p* = 0.002) were independent negative factors of treatment response, whereas clear tumor margin (OR = 4.043, *p* = 0.022) was a positive predicting factor of treatment response. Extrapancreatic origin tended to be a positive factor (*p* = 0.05) (Table 4).

**TABLE 4:** Univariate and multivariate logistic regression analyses for treatment response

	Univariate			Multivariate		
	OR	95% CI	*p*	OR	95% CI	*p*
Age	0.984	0.947–1.023	0.421			
Gender						
Male	Ref					
Female	0.460	0.181–1.173	0.104			
Functionality						
No	Ref					
Yes	0.875	0.258–2.963	0.830			
Primary site						
Pancreas	Ref			Ref		
Nonpancreas	2.480	0.991–6.202	0.052	3.123	0.998–9.778	0.050
Primary tumor resection						
No	Ref					
Yes	0.759	0.310–1.861	0.547			
Ki67	0.942	0.861–1.031	0.193			
≤10%	Ref			Ref		
>10%	0.262	0.090–0.762	0.014*	0.181	0.049–0.668	0.010*
Hepatic tumor burden						
<10%	Ref					
10%–25%	1.604	0.413–6.237	0.495			
25%–50%	1.108	0.303–4.050	0.876			
>50%	0.843	0.271–2.616	0.767			
Extrahepatic metastasis						
Bone	0.267	0.104–0.683	0.006*	0.149	0.045–0.489	0.002*
Lymph node	0.903	0.372–2.190	0.821			
Lung	0.392	0.062–2.486	0.320			
Others	1.008	0.259–3.933	0.990			
Hepatic tumor margin						
Unclear	Ref			Ref		
Clear	3.667	1.373–9.790	0.010*	4.043	1.225–13.344	0.022*
Arterial phase enhancement						
Without	Ref					
With	0.719	0.281–1.840	0.491			

* Indicates the significant differences of the results in Univariate and multivariate logistic regression models which used to analyze factors affecting treatment response. Ki‐67 >10%, Bone metastasis and clear hepatic tumor margin were factors with a *p* value of <0.10 in univariate analysis which included in the multivariate model. Differences with *p* < 0.05 were considered statistically significant.

### 3.5 Adverse events

None of the whole cohorts died during the perioperative period. The related postoperative adverse events were shown in Table 5. The adverse events caused by octreotide LAR were rare and mild. The most frequent adverse events that occurred after TAE were postembolization syndrome, including abdominal pain (58.6%), fever (53.4%), nausea and vomiting (29.3%), and transiently elevated liver enzymes (14.7%). Transient hypertension was found in 8 patients (8.7%). These symptoms were grade 1 and grade 2, which were tolerated and relieved within 1 week under proper symptomatic treatment. Biloma was found by imaging review in 2 patients without related symptoms. In addition, 3 patients developed liver abscesses and needed administration of antibiotics.

**TABLE 5:** Adverse events after transarterial embolization treatment

	Grade 1 (%)	Grade 2	Grade 3
Abdominal pain	37 (31.9)	31 (26.7)	0 (0)
Fever	49 (42.2)	13 (11.2)	0 (0)
Nausea and vomiting	30 (25.9)	4 (3.4)	0 (0)
Hypertension	23 (19.8)	2 (1.7)	0 (0)
Elevated liver enzymes	17 (14.7)	0 (0)	0 (0)
Hepatic abscess	0 (0)	0 (0)	3 (2.6)
Biloma	2 (1.7)	0 (0)	0 (0)

## 4 DISCUSSION

Extensive bilobar hepatic metastases could be found in approximately 65% NET patients at diagnosis,^8^ and there are little standard approaches and evidence in managing NETLM with extensive liver involvement. It is widely acknowledged that high liver tumor burden is an important factor linked to poor prognosis irrespective of treatment modality.^3^, ^7^, ^8^, ^9^, ^25^ In PROMID study, the median time to progression or tumor‐related death of patients treated with octreotide LAR dropped dramatically with liver involvement, being 29.4, 11.2, and 4.6 months in patients with liver involvement <10%, 10%–50%, and >50%, respectively.^9^ In our study, patients were all treated by TAE plus octreotide LAR and the median PFS of patients with HTB <10%, 10%–25%, 25%–50%, and >50% were 25.2, 13.6, 11.2, and 12.3 months, respectively. A general tendency of PFS decreasing with HTB could still be observed, but in patients with HTB >50%, the decline in PFS was not obvious. The PFS of patients with HTB >50% was at the same level as those with HTB 25%–50%. It is worth noting that 86% of patients enrolled in our study cohort were with ki67 >2% and 15% of patients were even with ki67 >10%, comprising a worse‐characterized population compared with the PROMID study cohort, in which 97% of patients were with ki67 up to 2%. And among the 35 patients with HTB >50% in our cohort, 19 patients presented with liver involvement even more than 75%. In this context, we still achieved comparable PFS in patients with HTB 10%–50% and, especially, remarkably prolonged PFS in patients with HTB >50%, indicating that patients with high HTB could benefit significantly from the combined treatment of TAE and octreotide LAR. The results of PROMID study pointed out that the antiproliferative response was more pronounced in patients with low hepatic tumor load and they tended to obtain a survival advantage from octreotide LAR treatment.^7^, ^9^ TAE is a widely accepted and effective option for reducing the tumor burden of NETLM.^17^, ^18^ The combined application of TAE and octreotide LAR aim to reduce HTB rapidly at the beginning of treatment, thus maximize the antiproliferative effect of octreotide LAR in a condition of relatively low HTB. In a retrospective study of Chen et al.,^25^ the hepatic PFS of patients with liver burden >50% was reported to be 9.5 months. However, more than half of the patients in Chen's study were free of systemic therapy after embolotherapy.^25^ From this perspective, systemic therapy, such as SSA, is also indispensable for managing NETLM.

In previous publications about TAE/TACE, the reported PFS/TTP ranged from 6.6 to 16.2 months, probably due to the difference in study populations.^17^, ^18^, ^19^, ^23^, ^26^ The study conducted by Ziv et al. reported that the median hepatic PFS was 6.6 months.^26^ Among the patients enrolled in his study, 27.5% were G3 or poor‐differentiated tumor and 57% had extrahepatic metastasis, which might be responsible for the relatively short PFS. Hur et al. reported a median PFS is 16.2 months from a study cohort with less extrahepatic metastasis (19.6%) and lower HTB (63% with hepatic burden ≤20%).^19^ The overall PFS was 13.6 months in the present study, slightly shorter than Hur et al.'s data. However, our cohort contained more aggressive patients including 86.2% G2 tumor, 63.8% with extrahepatic metastasis, and 50.9% with hepatic burden >25%. The results of the present study revealed that TAE plus octreotide LAR was more effective than the previous studies for patients with high HTB.

Apart from HTB, ki67 >10% and bone metastasis were also independent risk factors for progression. Ki67 or tumor grading is widely acknowledged to be an important factor for stratifying prognosis of patients with NET, for it directedly reflects the proliferative ability and aggressiveness of the tumor.^3^, ^5^, ^25^, ^26^, ^27^ WHO defined NETs with ki67 from 3% to 20% as G2 tumor.^2^ However, the range of ki67 for G2 is too wide to perform precise prognostic stratification. Nuñez‐Valdovinos et al. suggested that a ki‐67 cutoff of 10% had a particularly remarkable impact on the prognosis for G2 NETs.^28^ Our result helped confirm this opinion. Several previous publications have reported the presence of extrahepatic metastasis as a risk factor of prognosis,^19^, ^27^, ^29^ and the presence of bone metastasis was of high prognostic relevance.^3^, ^30^ In the GETNE‐TRASGU nomogram model, bone metastasis was one of the variables, indicating poor prognosis in patients with advanced NETs treated with SSA.^3^ Scharf et al. suggested that bone metastasis must be considered in the treatment of every individual NEN patient, irrespective of the primary site, grading or age of patients.^30^

The efficacy of TAE exerted different influences on patients with different HTB. In patients with low HTB, there were no significant differences in PFS observed between responders (CR/PR) and nonresponders (SD/PD), while in patients with high HTB (25%–50% and >50%), responders had markedly prolonged PFS compared with nonresponders, indicating that patients with high HTB could obtain significant prognostic advantage from effective TAE treatment. It could be speculated that for responders with high HTB, the absolute extent of reduction in tumor burden was more remarkable than those with low HTB. The ORR of our present study was 78.4%, consistent with the result of our previous publication.^20^ Compared with previous studies of TAE, in which ORRs were reported ranging from 37% to 72%,^13^, ^14^, ^15^, ^16^, ^17^, ^18^ the treatment efficacy that we have achieved by TAE plus octreotide LAR was favorable. From the multivariate logistic model for response, ki67, bone metastasis, and hepatic tumor margin were independent predictive factors, while HTB did not affect treatment response. Ki67 >10% and the presence of bone metastasis affected not only prognosis but also the response to TAE, indicating that decisions of TAE treatment should be made carefully for patients presenting with these factors. Tumor margin was reported to be associated with pathological grade in primary pancreatic NETs,^31^, ^32^ but no previous study sheds light on the relationship between the margin of NETLMs and the treatment efficacy of embolotherapy. In our study, a clear margin shown on liver lesions could predict better response to TAE. The underlying mechanism of this phenomenon is still unclear and warrants further study.

In the present study, we evaluated intrahepatic PFS and extrahepatic PFS, respectively, in the whole cohort and in patients with EHD. Since the results both showed that the intrahepatic PFS was markedly shorter than extrahepatic PFS, it is reasonable to assume that a NETLM patient's overall disease status depends largely on the status of liver disease instead of EHD even under the dual effects of systemic therapy and locoregional liver therapy. This result underlined the necessity of locoregional therapy for liver metastasis even if synchronic extrahepatic metastases were found. Arrese et al.^29^ demonstrated that patients with the EHD could still derive benefit from embolotherapy, but the influence of specific sites of EHD was not separately analyzed.

The combined treatment of TAE and octreotide LAR was safe and well tolerated. The most common adverse event after TAE was postembolization syndrome. The occurrence rate of postembolization syndrome reported in previous studies was 60%–80%.^14^, ^25^, ^33^ In our study, all the related symptoms after treatment could be relieved within 1 week. The presence of hepatic abscess was the most serious complication we have observed. The hepatic abscess might be developed from the injury and infection of biliary ducts which was caused by the embolization of peribiliary arterial plexus.^34^ Therefore, it is suggested that preexisting biliary duct dilation and previous surgery establishing cholangioenteric anastomosis should be deemed as relative contraindications for the embolotherapy. Once hepatic abscess was developed after TAE, timely application of antibiotics and percutaneous drainage were necessary.

To date, this is the first study assessing the efficacy and prognostic outcome of applying TAE plus octreotide LAR to manage patients with NETLM. Our previous publication showed that TAE plus SSA could safely and effectively reduce HTB of NETLM.^20^ The present study was conducted on the basis of the previous study and some of the results have confirmed the reproducibility. Still, there are some limitations. First, due to the retrospective nature and the consideration of sample size, it was impossible to make strict inclusion criteria. Thus, the presence of underlying bias was inevitable. Second, the follow‐up period was not long enough to observe the overall survival outcome. Nevertheless, the results shown in our study were encouraging and of clinical significance.

In conclusion, TAE plus octreotide LAR is effective in managing patients with NETLM. The PFS was remarkably prolonged in patients with HTB >50%. Selected patients with HTB >25% (ki67 ≤10%, absence of bone metastasis, clear tumor margin on contrast‐enhanced CT) could derive prognostic advantage from combined treatment of TAE and octreotide LAR.

## CONFLICT OF INTEREST

The authors declare no conflict of interest.

## AUTHOR CONTRIBUTIONS

Guarantors of the integrity of the entire study: Yu Wang, Jie Chen, and Minhu Chen; study concepts/study design: Yiming Liu, Haikuan Liu, Yu Wang, and Jie Chen; data acquisition: Haikuan Liu, Wenchuan Chen, and Hang Yu; data analysis/interpretation: all authors; manuscript drafting: Yiming Liu; manuscript revision: Yu Wang, Jie Chen, Yiming Liu, and Haikuan Liu; literature research: Yiming Liu, Haikuan Liu, Wenchuan Chen, and Hang Yu; clinical studies: Yu Wang, Jie Chen, Yiming Liu, Haikuan Liu, Wenchuan Chen, Hang Yu, Wang Yao, Wenzhe Fan, and Jiaping Li; agrees to ensure any questions related to the work are appropriately resolved: all authors; approval of the final version of the submitted manuscript: all authors.

## ETHICS STATEMENT

The study was approved by the Ethics Committee of the First Affiliated Hospital of Sun Yat‐sen Hospital.

## Data Availability

The data that support the findings of this study are available from the corresponding author upon reasonable request.
